# Redox responses in skeletal muscle following denervation

**DOI:** 10.1016/j.redox.2019.101294

**Published:** 2019-08-08

**Authors:** Mattia Scalabrin, Natalie Pollock, Caroline A. Staunton, Susan V. Brooks, Anne McArdle, Malcolm J. Jackson, Aphrodite Vasilaki

**Affiliations:** a MRC-Arthritis Research UK Centre for Integrated Research Into Musculoskeletal Ageing (CIMA), Department of Musculoskeletal Biology, Institute of Ageing and Chronic Disease, University of Liverpool, UK; b Department of Molecular and Integrative Physiology, University of Michigan, Ann Arbor, MI, USA

**Keywords:** Reactive oxygen species, Denervation, Skeletal muscle, Peroxide release, Hydrogen peroxide, Extensor digitorum longus, EDL, Glutathione, GSH, Glutathione peroxidase 1, GPx1, Hydrogen peroxide, H_2_O_2_, Heat Shock Proteins, HSPs, Manganese superoxide dismutase, MnSOD, Monoamine Oxidase A, MAO-A, NADPH oxidase 2, Nox2, NADPH oxidase 4, Nox4, Neuromuscular junction, NMJ, Oxidized glutathione, GSSG, Peroxiredoxin 6, Prx6, Phospholipase A2, PLA2, Phospholipase A2 (cytosolic isoform), cPLA2, Reactive oxygen species, ROS, Thioredoxin reductase 2, TrxR2, Tibialis anterior, TA, Wild type, WT

## Abstract

Previous studies have shown a significant increase in the mitochondrial generation of hydrogen peroxide (H_2_O_2_) and other peroxides in recently denervated muscle fibers. The mechanisms for generation of these peroxides and how the muscle responds to these peroxides are not fully established. The aim of this work was to determine the effect of denervation on the muscle content of proteins that may contribute to mitochondrial peroxide release and the muscle responses to this generation. Denervation of the tibialis anterior (TA) and extensor digitorum longus (EDL) muscles in mice was achieved by surgical removal of a small section of the peroneal nerve prior to its entry into the muscle. An increase in mitochondrial peroxide generation has been observed from 7 days and sustained up to 21 days following denervation in the TA muscle fibers. This increased peroxide generation was reduced by incubation of skinned fibers with inhibitors of monoamine oxidases, NADPH oxidases or phospholipase A2 enzymes and the muscle content of these enzymes together with peroxiredoxin 6 were increased following denervation. Denervated muscle also showed significant adaptations in the content of several enzymes involved in the protection of cells against oxidative damage. Morphological analyses indicated a progressive significant loss of muscle mass in the TA muscle from 7 days up to 21 days following denervation due to fiber atrophy but without fiber loss. These results support the possibility that, at least initially, the increase in peroxide production may stimulate adaptations in an attempt to protect the muscle fibers, but that these processes are insufficient and the increased peroxide generation over the longer term may activate degenerative and atrophic processes in the denervated muscle fibers.

## Highlights

• Skeletal muscle denervation results in mitochondrial generation of peroxides in muscle fibers.
• This increased peroxide generation was reduced by inhibitors of monoamine oxidases, NADPH oxidases or phospholipase A2 enzymes.
• The increased peroxide generation was associated with an increase in the muscle content of peroxiredoxin 6 and phospholipase A2 that may mediate phospholipid breakdown and are also potentially involved in activation of NADPH oxidase.
• The content of key antioxidant enzymes and Heat Shock Proteins involved in the protection against oxidative damage were also increased in muscle following denervation.
• Data are compatible with the proposal that the denervation-induced increase in mitochondrial peroxide production initially stimulates adaptive and repair processes in muscle, but sustained peroxide generation leads to degeneration and muscle atrophy.

## Graphical abstract

**Figure:**
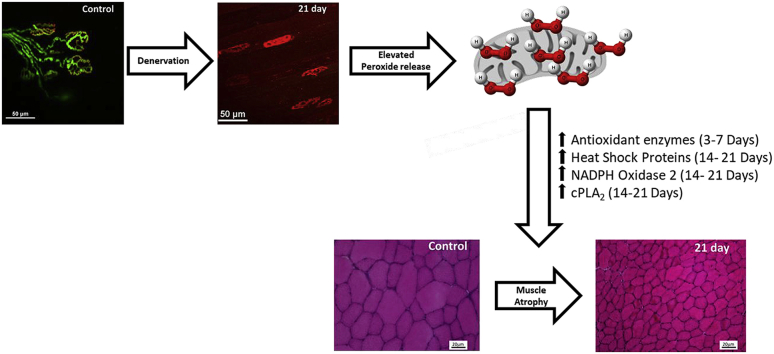

## 1 Introduction

Loss of muscle mass occurs during aging and is known as sarcopenia. This has a significant impact on health and is caused by several factors, among which impairment of the neuromuscular system appears to play a primary role. The neuromuscular system shows great plasticity and muscle fibers undergo a process of denervation and re-innervation repeatedly throughout life. With advancing age, the efficiency of this cycle appears to decline with a characteristic loss of neuromuscular junction (NMJ) structure (the synapse between a motor neuron and muscle fiber), motor units (the unit consisting of a single motor neuron and the muscle fibers it innervates), muscle mass (due to both the loss of motor units and individual muscle fiber atrophy) and together with an increase in neuronal axon sprouting with preferential re-innervation of slow-twitch muscle fibers [[1], [2], [3], [4]]. Denervated fibers atrophy and, on average, they are 35–50% smaller in comparison with innervated fibers in muscles of old rats [1]. However, the reasons for the age-related changes in muscle fiber denervation and disrupted NMJ innervation and function are not currently known.

A characteristic of aging is an increase of reactive oxygen species (ROS) activities that, if not adequately counteracted, can result in oxidative stress with decreased mitochondrial efficiency and cellular damage. Although numerous studies have highlighted the degenerative roles played by chronic increases in ROS [5], others have highlighted the signalling role played by ROS in response to alterations in redox homeostasis [2]. Thus, oxidants can activate and inactivate transcription factors, membrane channels and metabolic enzymes in addition to modulating calcium-dependent and phosphorylation signalling pathways [6].

Nerve transection models have been extensively used to investigate the mechanisms leading to rapid decline in muscle mass and function following the loss of innervation. Studies using this denervation model in rodents also point to a role of mitochondrial oxidative stress in the mechanisms of denervation-induced muscle atrophy [7,8,10].

Hydrogen peroxide (H_2_O_2_) plays a key role in cell signalling and is usually formed within the mitochondria by dismutation of superoxide generated from the electron transport chain. Beside the mitochondrial electron transport chain, up to 31 other enzymes have been implicated in the generation of H_2_O_2_ in skeletal muscle [9]. Studies performed by Muller et al. [7] indicated that H_2_O_2_ and other peroxides are produced in high amounts by muscle mitochondria [10] following short-term denervation and recent data published by our group show that this increased H_2_O_2_ may be generated by sources other than the electron transport chain within mitochondria [8].

The aim of the present study was to determine the effect of prolonged denervation on peroxide release and proteins that regulate redox homeostasis in muscle fibers.

## 2 Materials and methods

### 2.1 Mice

8–10 month old male C57BL/6 WT and B6. Cg-Tg (Thy1-YFP) mice were used for this study, with 4 mice used per time point (unless otherwise stated). The C57BL/6 WT mice were used for biochemical analyses whereas the B6. Cg-Tg (Thy1-YFP) mice were used for histological analyses and NMJ visualization as previously described [8]. Mice were fed ad libitum on a standard laboratory diet, subjected to a 12-h light/dark cycle and maintained under SPF conditions. All experiments were performed in accordance with UK Home Office guidelines under the UK Animals (Scientific Procedures) Act 1986 and received ethical approval from the University of Liverpool Animal Welfare Ethical Review Body (AWERB).

### 2.2 Surgical denervation of the tibialis anterior (TA) and extensor digitorum longus (EDL) muscles

Mice underwent surgical denervation of the tibialis anterior (TA) and extensor digitorum longus (EDL) muscles as described by Pollock et al. [8]. Briefly, mice were anesthetized with Isoflurane 0.2% (ISO) while Buprenorphine (analgesic; 1 mg/kg, SC 0.1 ml) was administered before surgery. To induce denervation of the TA and EDL muscles, a small section of the peroneal nerve was removed. The external wound was sutured and mice were maintained under close observation until recovery from anesthetic. Mice were then sacrificed at different time points after denervation (1, 3, 7, 14, 21 days) through cervical dislocation and TA and EDL muscles were immediately removed.

### 2.3 Assessment of morphological changes in muscle and nerve

TA muscles were mounted directly on a cork disk, surrounded with O·C.T. mounting medium (Thermo Fisher Scientific, Waltham, MA, USA), frozen rapidly in isopentane cooled in liquid nitrogen and sectioned (12 μm) using a cryostat (Leica CM1850, Leica, Wetzlar, Germany) as described by Vasilaki et al., [11]. To assess fiber cross-sectional area (CSA) transverse sections were fixed onto slides in ice-cold methanol and stained with Alexa Fluor 488 nm conjugated wheat germ agglutinin (WGA-488) (5 μM) (Vectorlabs, Burlingame, CA, USA). Multiple images were collected by confocal microscopy (Nikon, Kingston, UK) using ×20 objective to capture the section in its entirety. The images were collated into a montage and analyzed using ImageJ software (U.S. National Institutes of Health, USA). Separate transverse sections of TA muscle were stained with hematoxylin and eosin (H&E) using a standard protocol [12]. Each slide was examined using a Nikon Ci microscope (Nikon Kingston, UK) equipped with ×4 and ×20 objectives.

In order to assess the loss of innervation at different time points the EDL muscle from Thy1-YFP mice was pinned onto sylgard plates (Fernell, Preston, UK) and fixed in 10% neutral buffered formalin. Whole EDL muscles were incubated (30 min) with bungarotoxin conjugated with Alexa Fluor-532nm (10 μg/ml) and Dapi (1:500) (Invitrogen, Paisley, United Kingdom). Nerves were visible due to the expression of YFP. Muscles were imaged via confocal microscopy (Nikon A1, Kingston, UK) using a water immersion lens.

### 2.4 Analysis of mitochondrial peroxide generation

Analysis of the mitochondrial release of H_2_O_2_ (and other peroxides) from the TA muscle was performed by following the oxidation of amplex red as described by Pollock et al. [8]. Small bundles of muscle fibers were placed into 200 μM saponin (Sigma Aldrich, UK) in relax solution (containing 4.5 mM MgATP, 1 mM Free Mg, 10 mM Imidazole, 2 mM EGTA and 100 mM KCl, pH 7.0) to permeabilize the fibers. Saponin was washed from the tissue through 3 cycles of fresh relax solution with gentle agitation prior to placing the bundles into amplex red solution (containing 37.5U/ml SOD, 19.44 mM Amplex red, 5U/ml Horse radish Peroxidase (HRP) in ROS buffer (125 mM KCL, 10 mM Hepes, 5 mM MgCl_2_, 2 mM K_2_HPO_4_) in black 96 well plates (Corning, Wiesbaden, Germany). Bundles were then incubated at 37 °C with amplex red solution without added substrates to assess state 1 peroxide production, or in the presence of specific enzyme inhibitors: (20 μM AACOCF3 - phospholipase A2 inhibitor; 0.5 mM apocynin – a non specific NADPH oxidase inhibitor; 100 μM chlorgyline - monoamine oxidase A inhibitor; 100 μM pargyline - monoamine oxidase B inhibitor) as described in Pollock et al. [8]. The fluorescent signal was recorded at 590 nm using a fluorimeter (Fluorstar, BMG Labteck, Germany).

### 2.5 Muscle content of total and oxidized glutathione

TA muscles were ground under liquid nitrogen and the resulting powder was added to 1% sulfosalicylic acid. Following homogenization, samples were centrifuged for 10 min at 14,000g and the supernatant was collected for total and oxidized glutathione analyses as described by Vasilaki et al. [13]; whilst the pellet was re-suspended in 0.5 M Tris/HCl buffer (pH 7.6) for total protein content determination [14]. The automated glutathione recycling method described by Anderson [15] was used to assess the total glutathione content using a 96-well plate reader (SPECTROstar Nano, BMG labtech, Ortenberg, Germany). To determine the oxidized glutathione content, samples were firstly derivatized with 2-Vinylpyridine and analyzed as above.

### 2.6 SDS-PAGE and immunoblotting

Denervated and control TA muscles were ground in liquid nitrogen. The resulting powder was added to 1% SDS (Sigma-Aldrich Ltd, Gillingham, Dorset, United Kingdom) containing protease inhibitors (Thermo Fisher Scientific, Waltham, MA, USA) and homogenized for 30–60 s. Samples were centrifuged for 10 min at 13,000g, the supernatant collected and protein content analyzed using a BCA assay kit (Thermo Fisher Scientific, Waltham, MA, USA). Thirty micrograms of total protein was applied to a 12% polyacrylamide gel with a 4% stacking gel. The separated proteins were transferred onto nitrocellulose membranes by western blotting as previously described by Sakellariou et al. [16]. Ponceau red (Sigma-Aldrich, St. Louis, MI, USA) was used to verify the effectiveness of transfer procedure and to normalize the results as described by Romero-Calvo et al. [17]. The membranes were analyzed using antibodies against; Nox2/gp91 phosphorylated (Nox2/gp91^phox^), Nox4, p22 phosphorylated (p22^phox^), p40 phosphorylated (p40^phox^), p47 phosphorylated (p47^phox^) and p67 phosphorylated (p67^phox^), Ras-related C3 botulinum toxin substrate 1 (Rac-1), peroxiredoxin 6 (Prx6), cytosolic phospholipase A2 isoform (cPLA2), monoamine oxidase A (MAO-A), thioredoxin reductase 2 (TrxR2), glutathione peroxidase 1 (GPx1), manganese superoxide dismutase (MnSOD; SOD2) and Heat Shock Proteins (HSPs) Hsp25, Hsc70 and Hsp70 (Table 1, Table 2). Chemiluminescence was detected using the ChemiDocTM imaging system (Bio-Rad, Hercules, CA, USA) following addition of ECL (Bio-Rad, Hercules, CA, USA). Densitometry analysis was performed using ImageJ software according to the protocol described by McLean [18].

**Table 1:** List of the antibodies utilized for SDS-PAGE Western blot analysis.

Target	Company	Code	Clonal	Host species	Dilution
GPx1	Abcam	ab22604	Polyclonal	Rabbit	1:1000
Hsp25	ENZO	801d	Polyclonal	Rabbit	1:1000
Hsp70	Abcam	ab31010	Polyclonal	Rabbit	1:1000
Hsc70	Stress Gen	SPA815	Monoclonal	Rat	1:750
MAO-A	Abcam	ab126751	Monoclonal	Rabbit	1:1000
Nox4	Abcam	ab133303	Monoclonal	Rabbit	1:1000
Nox2/gp91phox	Abcam	ab43801	Polyclonal	Mouse	1:500
P22 phox	SCBT	sc271968	Monoclonal	Mouse	1:500
P40 phox	SCBT	sc48388	Monoclonal	Mouse	1:500
P47 phox	SCBT	sc17845	Monoclonal	Mouse	1:500
P67 phox	BD Bioscience	610913	Monoclonal	Mouse	1:500
cPLA2	CST	2832S	Polyclonal	Rabbit	1:1000
Prx6	Abcam	ab133348	Monoclonal	Rabbit	1:1000
Rac-1	Abcam	ab33186	Monoclonal	Mouse	1:500
MnSOD	CST	13194S	Monoclonal	Rabbit	1:1000
TRxR2	CST	12029S	Monoclonal	Mouse	1:1000

**Table 2:** List of the secondary antibodies utilized for SDS-PAGE Western Blot analysis.

Secondary Antibody	Company	Code	Dilution
Anti-mouse IgG, HRP-linked	CST	7076S	1:5000
Anti-rabbit IgG, HRP-linked	CST	7074S	1:5000
Anti-rat IgG, HRP-linked	CST	7077S	1:5000

### 2.7 Analysis of protein carbonylation

Denervated and control TA muscles from C57/Bl6 mice were homogenized in Hepes buffer and analyzed using the *OxyBlot* protein oxidation detection kit (Merk, Darmstadt, Germany). Samples were separated on 12% polyacrylamide gels, transferred on a PVDF membrane (GE Healthcare Bio-Sciences, Pittsburgh, USA) and analyzed as specified. Densitometry analysis of the bands at different molecular weights was performed using ImageJ software.

### 2.8 Statistical analysis

Statistical analysis was performed using IBM SPSS statistic version 22 software (IBM analytics, New York, USA). All tests have been carried out with a 95% confidence interval and the level of significance was set at 0.05. Normal distribution was verified using the Shapiro-Wilk test and the data were expressed as the mean ± standard error mean. One-way ANOVA was performed using Bonferroni's correction to identify significant differences between the control group and the other time points. For those variables where the assumption of normal distribution was violated, differences within and between groups were determined by Kruskal-Wallis test followed by Mann-Whitney *U* test for post hoc analysis applying Bonferroni's correction.

## 3 Results

### 3.1 Morphological and structural changes following denervation

**Fig. 1:**
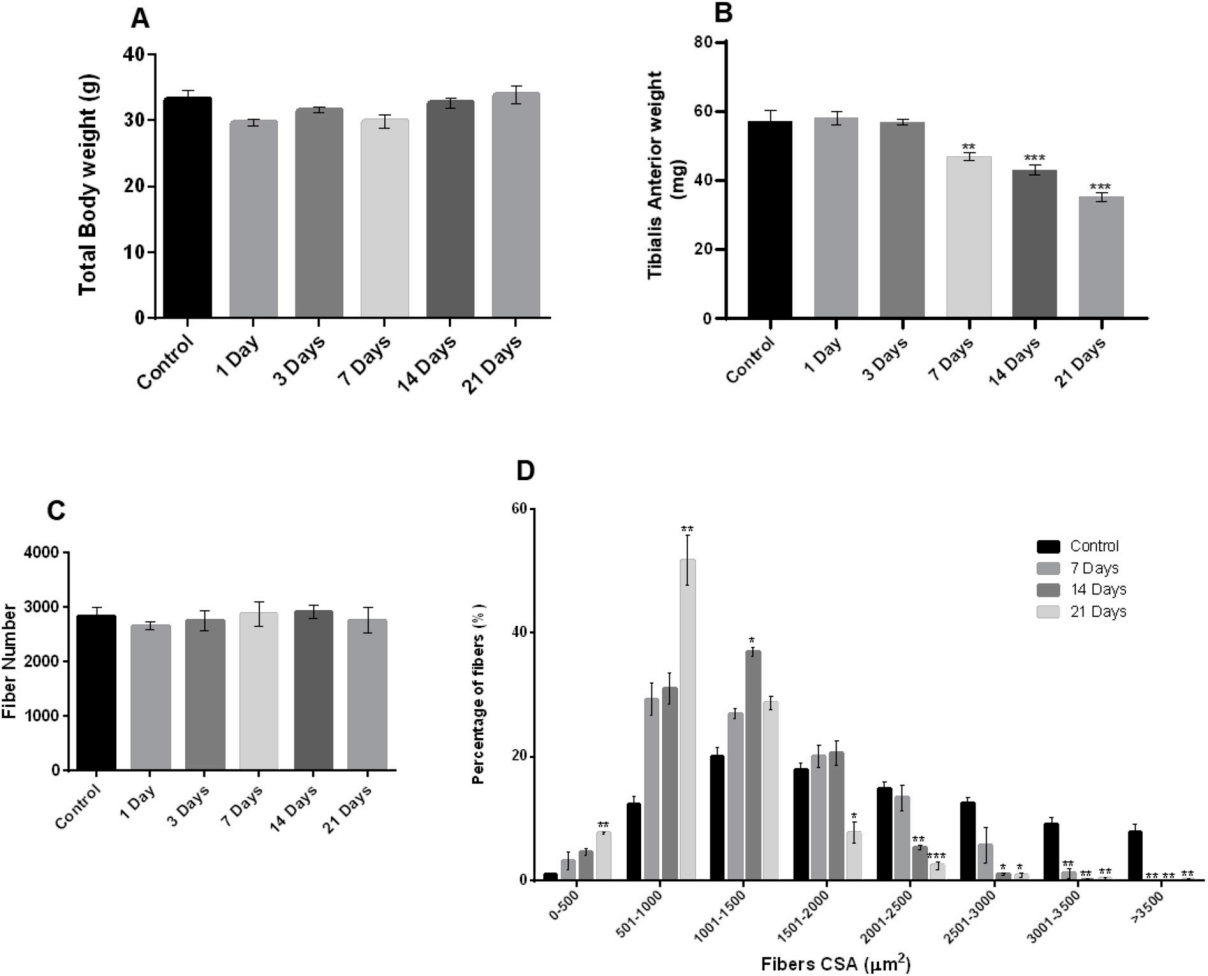
(A) Total body weights (B) TA muscle weights (C) fiber number and (D) fiber CSA from control mice (non-denervated) and mice at 1, 3, 7, 14 and 21 days post-denervation. Histograms represent the mean and the standard error of the mean for each experimental group (n = 4). *p < 0.05 - **p < 0.01 - ***p < 0.001 compared with the control group.

No significant difference in body weights of mice were observed between the control and the denervated groups (Fig. 1A). Analyses indicated a progressive and significant loss of mass in the TA muscle from 7 days up to 21 days following denervation (F (5,19) = 33.204 p < 0.01) (Fig. 1B) which was not associated with any loss of fiber number (F (5,10) = 0.316 p > 0.05) (Fig. 1C) but appeared entirely due to fiber atrophy (Fig. 1D). Analysis of the distribution of fiber CSA indicated that denervation induced a decrease in the number of larger fibers at 7, 14 and 21 days post-nerve transection with an increase in the number of smaller fibers compared with control muscle (Fig. 1D).

**Fig. 2:**
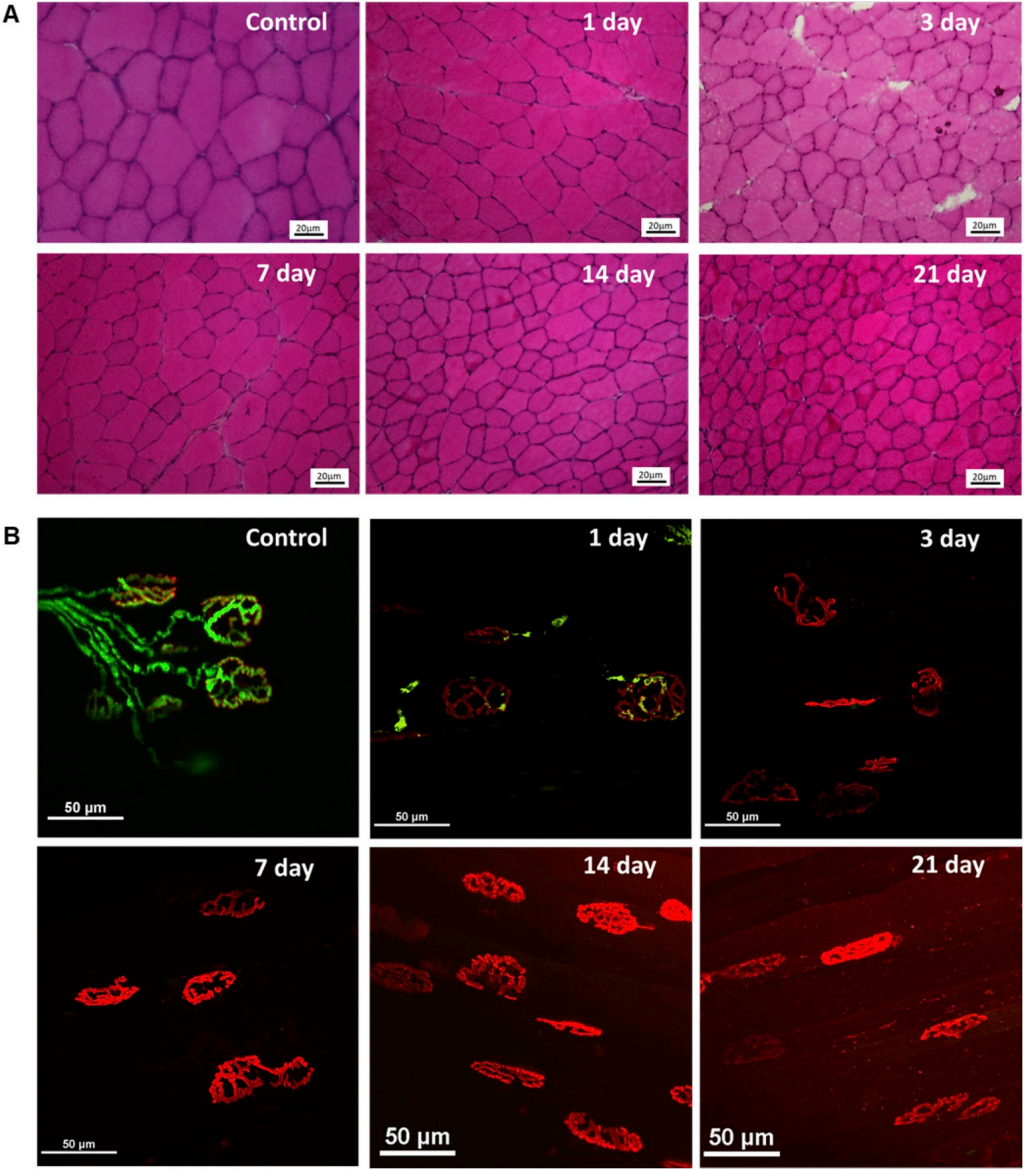
(A) Cross-sections of TA muscles visualized by H&E staining and (B) NMJs observed in whole mount EDL muscles from control mice (non-denervated) and mice at 1, 3, 7, 14 and 21 days post-denervation. Images show the peripheral nerves (green) and acetylcholine receptors (red). (For interpretation of the references to colour in this figure legend, the reader is referred to the Web version of this article.)

While the decline in fiber size reported in Fig. 1 was clearly evident, histological examination revealed no structural abnormalities within muscles (i.e. presence of necrotic or regenerating fibers; Fig. 2A). No significant infiltration of the muscle by immune or other mononuclear cells was apparent following denervation with hematoxylin and eosin staining (Fig. 2A). Since this was not within the scope of the study, specific staining for potential invading cells was not performed and therefore, it is not possible to entirely rule out the possibility that increased numbers of immune cells were present at some time points.

Examination of the EDL muscles confirmed that neuronal input to the NMJs was lost by 3 days post-nerve transection [8]. There was no evidence of re-innervation at 14 or 21 days and acetylcholine receptors (stained using α-bungarotoxin) remained present in characteristic clusters. Further bungarotoxin-staining distant from NMJs was also apparent along the length of the muscle fibers by 21 days post-denervation (Fig. 2B).

### 3.2 Peroxide production by mitochondria in bundles of TA muscle fibers in state 1

**Fig. 3:**
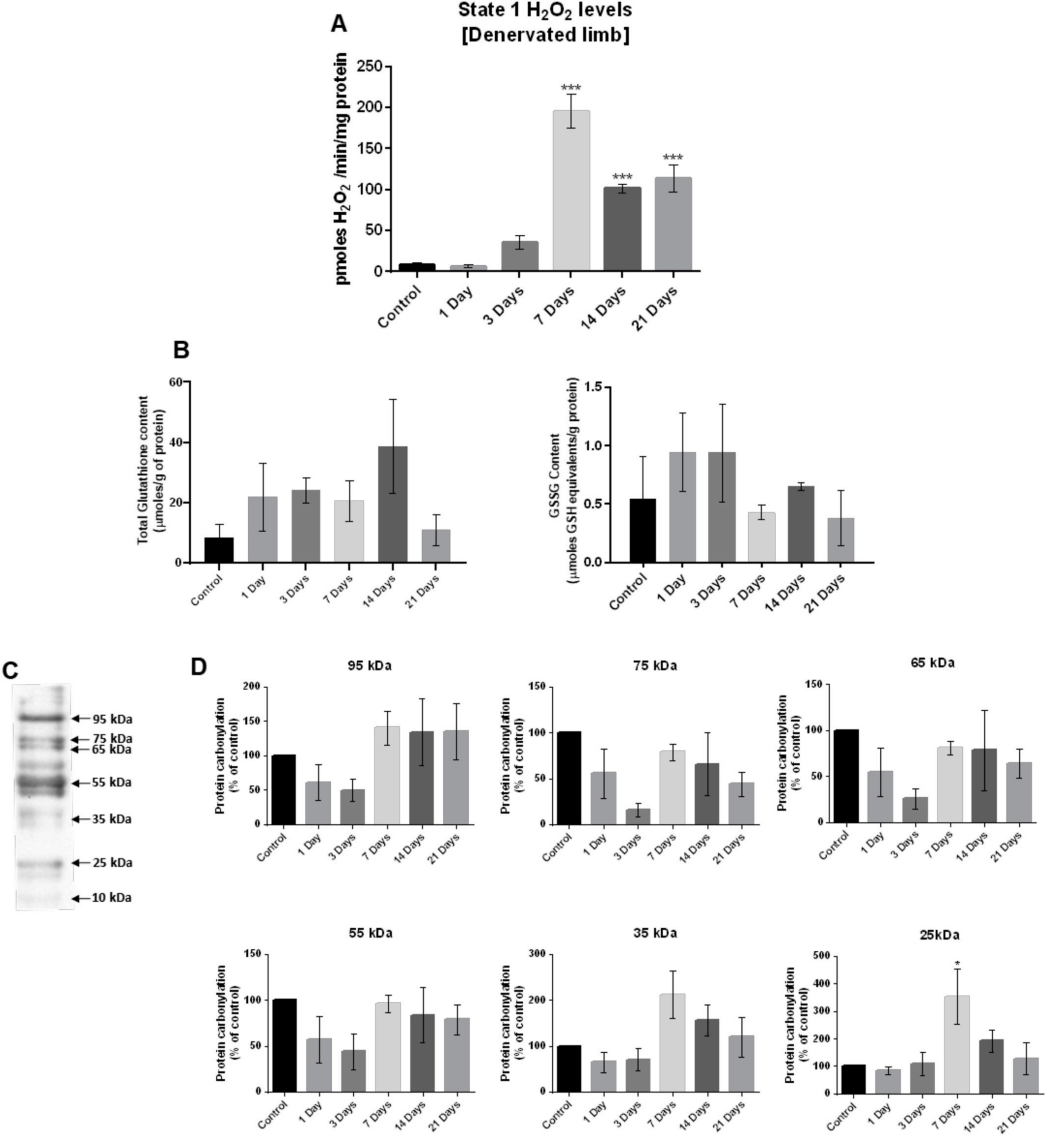
(A) State 1 H_2_O_2_ production in mitochondria from permeabilized fibers from the TA muscles determined by oxidation of amplex red. (B) Total glutathione (GSH) and oxidized glutathione (GSSG) contents in TA muscles from control (non-denervated) mice and mice at 1, 3, 7, 14 and 21 days post-denervation. (C) A representative lane of an *OxyBlot* for control muscle showing the bands detected with approximate molecular weights (MW) indicated. (D) Quantification of protein carbonyls by densitometric analysis of the protein bands at different molecular weights. Histograms represent the mean and standard error of the mean for each experimental group (n = 4). *p < 0.05 - **p < 0.01 - ***p < 0.001 compared with the control group.

A statistically significant increase in the rate of oxidation of amplex red was observed from bundles of permeabilized TA fibers following denervation (F (4,11) = 44.354 p < 0.001) (Fig. 3A). Peak mitochondrial peroxide production in state 1 was seen at 7 days following denervation although levels remained elevated up to 21 days after denervation (Fig. 3A).

### 3.3 Total and oxidized glutathione content of muscle

The total glutathione (GSH) and oxidized glutathione (GSSG) contents of denervated TA muscles are shown in Fig. 3B. Data show no significant differences in total GSH (F (5,16) = 1.473 p > 0.05) or GSSG (F (5,15) = 1.291 p > 0.05) content following denervation.

### 3.4 Protein oxidation in TA muscles following denervation

Analysis of protein carbonylation has been widely used as a measure of oxidative damage to proteins. The *OxyBlot* approach is relatively non-specific and semi-quantitative but potentially provides insight into the molecular mass of oxidized protein targets. Soluble proteins from denervated and control TA muscles were separated by SDS-PAGE and were analyzed for carbonyl content following western blotting. An illustrative blot of the pattern of bands is shown in Fig. 3C. Six major bands of approximate molecular weight 95, 75, 65, 55, 35, and 25 kDa were quantified by band densitometry (Fig. 3D). Densitometry analysis revealed no major differences in the carbonyl content between control and denervated muscles except from the band seen at approximately ∼25 KDa, which showed increased intensity at 7 days following denervation (F (5,17) = 3.616 p < 0.05) (Fig. 3D) when the significant increase in mitochondrial peroxide generation from the denervated muscle fibers was also evident (Fig. 3A).

### 3.5 Potential sources of H_2_O_2_ in TA muscle following denervation

**Fig. 4:**
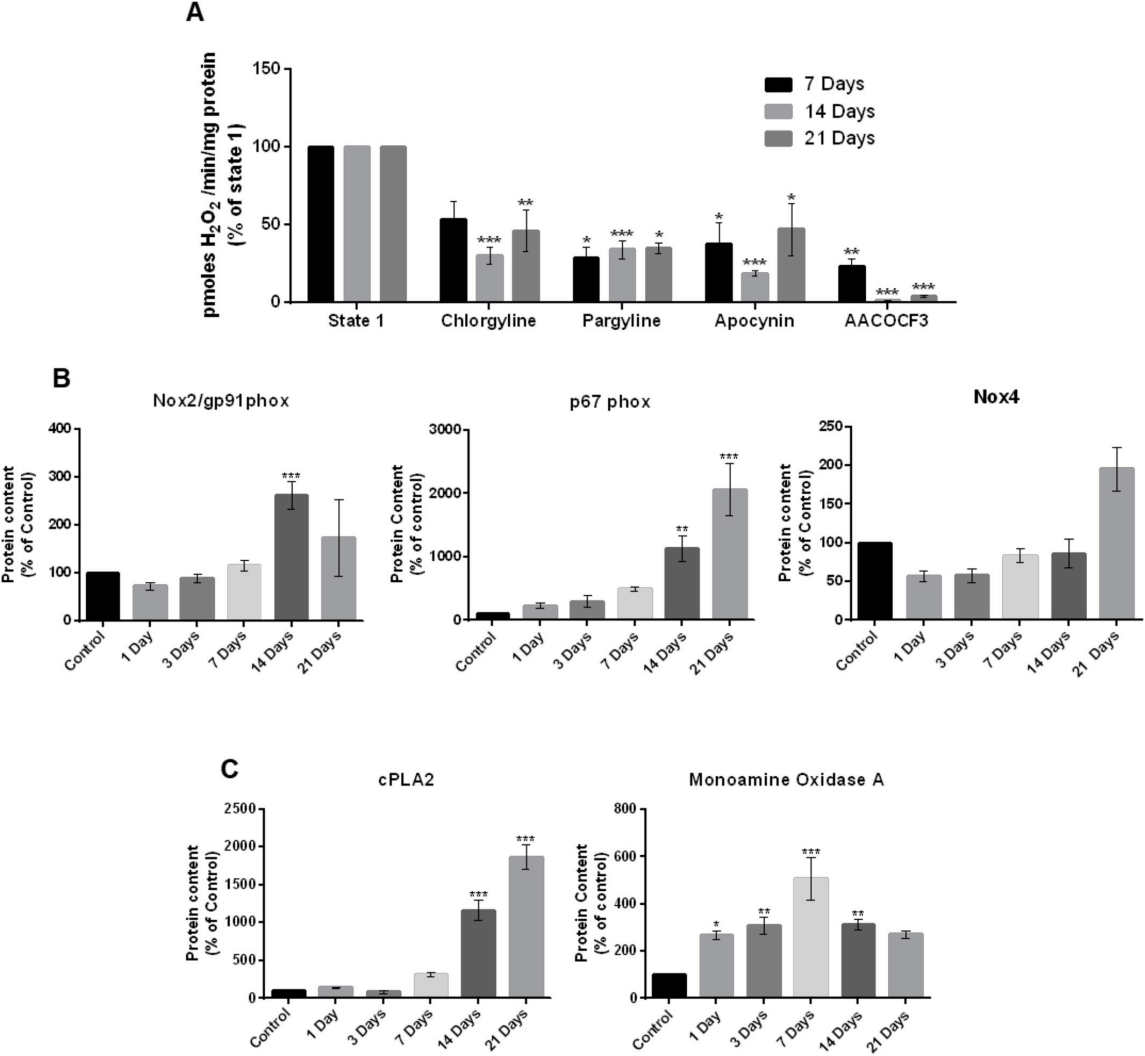
(A) State 1 mitochondrial H_2_O_2_ generation from permeabilized fibers of the TA muscle in the presence of different enzyme inhibitors. Addition of each inhibitor (chlorgyline (monoamine oxidase A), parglyline (monoamine oxidase B), apocynin (NADPH oxidase), AACOCF3 (PLA2) resulted in significantly reduced H_2_O_2_ generation compared to untreated fiber bundles at 14 and 21 days post-denervation. Histograms represent the mean and standard error of the mean for each experimental group (n = 4). *p < 0.05 - **p < 0.01 - ***p < 0.001 compared with its state 1. (B) Quantification of western blots of NADPH oxidase 2 subunits (Nox2/gp91^phox^ and p67^phox^) and NADPH oxidase 4 (mitochondrial isoform) and (C) of cPLA2 and monoamine oxidase A content in TA muscle from control mice (non-denervated) and mice at 1, 3, 7, 14 and 21 days post-denervation. Histograms represent the mean protein content and standard error of the mean for each experimental group (n = 4). *p < 0.05 - **p < 0.01 - ***p < 0.001 compared with the control group.

In order to determine potential sources of H_2_O_2_ production in TA muscles in state 1 following denervation, permeabilized muscle fiber bundles were incubated with specific enzyme inhibitors during analysis of peroxide generation as previously described [8]. All inhibitors reduced peroxide release to variable extents at the different time points examined (7, 14 and 21 days post-denervation) (Fig. 4A).

Functionally active NADPH oxidase 2 (Nox2) is a multimeric enzyme composed of various subunits: Nox2/gp91^phox^ and p22^phox^ comprise the membrane-bounded part of the enzyme whereas, p47^phox^, p67^phox^, p40^phox^, and Rac-1 subunits organize and assemble the functional complex and regulate its activity [[19], [20], [21]]. The muscle content of all of the Nox2 subunits examined increased after denervation, although the relative increases differed between subunits in terms of timing and magnitude of change. While most of the subunits showed changes that could be quantified by densitometry within the dynamic range of western blots, some of the regulatory subunits were undetectable, or barely detectable in the baseline (non-denervated) samples and in these cases only example blots are shown in the supplementary data to provide examples of the changes observed. Nox2/gp91^phox^ content (F (5,14) = 10.309, p < 0.01, Fig. 4B) increased with time to an apparent peak at 14 days post-denervation and p67^phox^ protein content showed a significant increase compared to control at 14 days after denervation which increased further at 21 days post denervation (F (5,17) = 15.595, p < 0.01).

Example blots showing the increased p40^phox^ content are presented in Supplementary data 1A. The p22^phox^ content was significantly increased at 7, 14 and 21 days post-denervation whereas p47^phox^ protein content was significantly increased at 3 days following denervation (F (5,15) = 7.390, p < 0.01; Supplementary data 1B). Rac-1 is reported to trigger activation of the NADPH oxidase complex and Rac-1 content showed a statistically significant increase at 7 days (F (5,16) = 3.829, p < 0.01) post-denervation (Supplementary data 1B).

The muscle content of NADPH oxidase 4 (Nox4) showed little change up to 14 days post-denervation compared with control and although a trend towards an increase was seen at 21 days, this did not reach statistical significance when compared with the control group (Fig. 4B).

The protein levels of three other proteins that might potentially be involved in generation of fatty acid peroxides or H_2_O_2_ were examined. The muscle content of cPLA2 increased post-denervation and was significantly higher than control at 14 and 21 days (F (5, 16) = 98.569, p < 0.01) (Fig. 4C). Monoamine oxidase A protein content was significantly increased at 1 day following denervation and remained increased up to 14 days following denervation (F (5, 16) = 10.986, p < 0.01) (Fig. 4C). Prx6 is a peroxiredoxin that may also have PLA2 activity under some conditions and although this protein was undetectable in control muscle samples, a clear increase was seen in muscle up to 21 days post-denervation (Supplementary data 2A).

### 3.6 Muscle content of mitochondria-localized regulatory enzymes following denervation

**Fig. 5:**
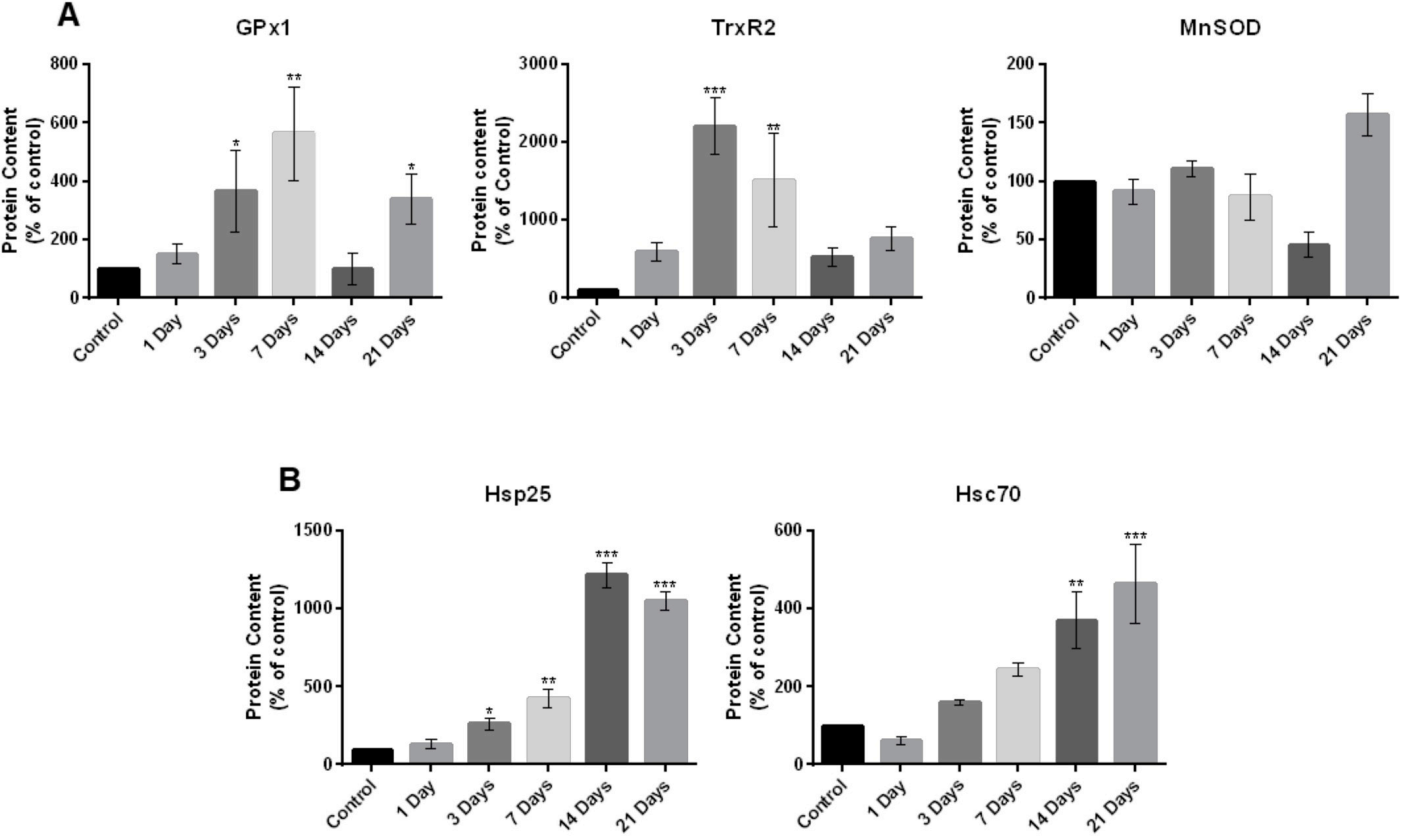
(A) Quantification of western blots for mitochondrial antioxidant enzymes (GPx1, TRxR2, MnSOD) and (B) HSP (Hsp25, Hsc70) content in TA muscle from control mice (non-denervated) and mice at 1, 3, 7, 14 and 21 days post-denervation. Histograms represent the mean protein content and standard error of the mean for each experimental group (n = 4). *p < 0.05 - **p < 0.01 - ***p < 0.001 compared with the control group.

The content of three major mitochondrial enzymes involved in redox regulation were analyzed: The MnSOD content in muscle did not change significantly following denervation (Fig. 5A), but TrxR2 was significantly increased at 3 and 7 days post denervation and then declined over the following time points (F (5,15) = 10.899 p < 0.01), while the GPx1 content was significantly increased at 3, 7 and 21 days following denervation (Fig. 5A).

### 3.7 HSP content of TA muscles following denervation

Data in Fig. 5B show that skeletal muscles from adult mice also responded to denervation by an increase in the content of some HSPs. Hsp25 was significantly increased at 3 days and continued to increase at 7, 14 and 21 days following denervation ( F (5, 16) = 147.142, p < 0.01). The Hsc70 content of denervated TA muscles was significantly increased at 14 and 21 days post-denervation ( F (5, 15) = 16.073, p < 0.01). Hsp70 was undetectable in some control muscle samples but appeared to increase at 14 and 21 days post-denervation (Supplementary data 2B).

## 4 Discussion

It is now well established that during aging a number of muscle fibers lose functional innervation, leading to significant fiber degeneration and atrophy [1,3] and studies using nerve transection models in rodents have indicated a role for mitochondrial oxidative stress in the mechanism of denervation-induced muscle atrophy [7,8,10]. Specifically, previous work from our group and others has shown that denervation of muscle leads to a large increase in the release of H_2_O_2_ and lipid peroxides from muscle mitochondria over a short time course [7,8,10]. In this study, we extended the time course of denervation in order to examine the longer-term effect of muscle denervation on peroxide release and proteins that regulate redox homeostasis in muscle fibers.

Full denervation of the TA muscle was achieved by surgically removing a small section of the peroneal nerve prior to entry into the muscle. Morphological analyses of the TA muscle indicated a progressive significant loss of muscle mass from 7 days up to 21 days post-denervation. This occurred without loss of fibers (Fig. 1C) and hence was due to fiber atrophy (Fig. 1D). Our previous published data have demonstrated that by 7 days following denervation there was complete loss of pre-synaptic axonal input at the NMJs [8] and our current data confirm this (Fig. 2B) and demonstrate the longer-term effects on muscle fiber atrophy (Fig. 1).

The extended time course of mitochondrial peroxide generation reported here showed a significant increase in H_2_O_2_ generation from fiber bundles by 7 days post-denervation, which was sustained up to 21 days (Fig. 3A). Pollock et al. [8] previously demonstrated by addition of substrates and inhibitors of the electron transport chain that the increased peroxide generation by muscle mitochondria post-denervation did not derive from the electron transport chain but indicated that alternative sources of peroxides were likely to be involved. Skeletal muscle mitochondria contain several other potential sources of ROS including monoamine oxidases [22]), NADPH oxidase [16] and phospholipases [23] and inhibitor studies have suggested that all of these proteins may be involved in the denervation-induced increase in mitochondrial ROS ([8] and Fig. 4A).

Monoamine oxidases are flavoenzymes that are present in the mitochondrial outer membrane [24] and exist in two isoforms, monoamine oxidase A and B that share approximately 70% of sequence identities but are distinguished by different substrate specificity [25]. Both monoamine oxidase A and B play a primary function in regulating neurotransmitter levels and the degradation of amines [24]. Skeletal muscle has been reported to contain both monoamine oxidase A [26] and B [27] and addition of chlorgyline (monoamine oxidase A) and pargyline (monoamine oxidase B) inhibitors reduced the oxidation of amplex red by permeabilized muscle fibers. Monoamine oxidase A protein content was also found to be increased prior to the increase in mitochondrial peroxide release (Fig. 4C) whereas monoamine oxidase B protein content was unchanged (data not shown).

Previous studies have shown that, following denervation, lipid peroxides are also released from mitochondria and can oxidize amplex red [8,10]. Our current inhibitor data supports this possibility since addition of the cPLA2 inhibitor, AACOCF3, reduced mitochondrial peroxide production (Fig. 4A) and muscle levels of cPLA2 were also increased post-denervation (Fig. 4C and [8]).

Other studies have demonstrated that skeletal muscles contain different isoforms of NADPH oxidase [16,28]. Addition of apocynin (a non-specific NADPH oxidase inhibitor) significantly reduced the denervation-induced mitochondrial amplex red oxidation at all time points following denervation (Fig. 4A), but the cPLA2 inhibitor AACOCF3 and the monoamine oxidase inhibitors also caused a marked reduction of peroxide release. These apparently conflicting pieces of data may potentially be explained by a lack of specificity of the inhibitors used or by involvement of complex interacting pathways, such as for instance, a potential direct role for PLA2 in the production of peroxide together with a role for PLA2 in activation of NADPH oxidase [29,30].

In the present study, we examined two NADPH oxidase isoforms that are reported to be present in skeletal muscle, Nox4, present within mitochondria, and Nox2 which is found mainly in the cytosolic environment [28]. Due to the reported sub-cellular location of the two isoforms, only Nox4 could likely contribute to the post-denervation increase in mitochondrial peroxide generation, but the muscle content of Nox4 showed no significant changes throughout the time course (Fig. 4B). However, our study focused on measuring protein content and not activity, therefore we cannot rule out altered activity of Nox4.

In contrast, significant post-denervation increases in muscle were seen in the content of components of the Nox2 complex. All Nox2-related subunits were seen to increase to some extent after denervation (Fig. 4B and Supplementary data 1). The interaction of Nox2/gp91^phox^ with p22^phox^ is a necessary step for the assembly of the Nox2 complex and the content data presented suggest that this would be maximal from 14 days following denervation. The protein content of p47^phox^ and Rac-1 were significantly increased between 3 and 7 days post-denervation (Supplementary data 1). Phosphorylation of p47^phox^ and Rac-1 activation have previously been reported in skeletal muscle and these post-translational events lead to translocation and binding of cytosolic subunits to the cell membrane and Nox2 and p22^phox^ to form a functionally active complex [21]. The content of p67^phox^ and p40^phox^ subunits was also increased at 14 days post-denervation. These are reported to be mainly cytosolic and translocate to the cell membrane upon Nox2 activation [21].

NADPH oxidases can be activated by different stimuli and different pathways can induce their assembly. It has previously been shown that PLA2 activity can activate NADPH oxidases [29] and can promote ROS generation in muscle mitochondria [31] and cytosol [32]. PLA2 is mainly involved in the hydrolysis of sn-2 fatty acyl chains of phospholipids generating fatty acids such as arachidonic acid which are important substrates for cyclooxygenases (COX) and lipoxygenases (LOX) and involved in the activation of NADPH oxidase and generation of ROS [33]. In the present study, an increase in cPLA2 content was seen at 14 days following denervation which would be compatible with a potential role for this enzyme in Nox2 activation. Previously published data has shown that cPLA2 is a negative regulator of growth, specifically of striated muscle and deletion of cPLA2 promotes striated muscle growth [34]. Our data demonstrated that the cPLA2 content was significantly increased in muscle at 14 days post-denervation and remained increased thereafter (Fig. 4C) when muscle fiber atrophy was evident.

Prx6 protein content was also increased in muscle at 3 days post-denervation (Supplementary data 2A). This protein is an atypical peroxiredoxin, which has potential PLA2 activity [35]. It is a 1-Cys peroxiredoxin with unique bi-functional characteristics and can translocate into the mitochondria during stress conditions where it plays a primary role in binding and reducing phospholipid hydroperoxides [36]). This protein also acquires PLA2 activity if it is over-oxidized [37]. This suggests the possibility that the increased peroxide release from mitochondria may induce the translocation of Prx6 into mitochondria where it becomes over-oxidized and undergoes conformational changes acquiring PLA2 properties. These conformational changes allow Prx6 to produce fatty acids, including arachidonic acid that have been reported to induce the phosphorylation of p40^phox^, p47^phox^ and p67^phox^ and their subsequent translocation to the plasma membrane allowing the assembly of Nox2 [38].

Thus, overall, the data obtained are compatible with the hypothesis that, following denervation, muscle mitochondria increase peroxide release and this leads to an adaptive increase in Nox2 content and that this enzyme may be activated via Prx6-PLA2-mediated signalling pathways. It is important to note that the increases in muscle content of Nox2 protein and its regulatory sub-units are unlikely to have contributed to the observed increase in mitochondrial peroxide generation post-denervation. Previous studies have not identified Nox2 in muscle mitochondria [16]. Rather these changes are likely to reflect an additional adaptation of the muscle to denervation. We have previously speculated that a key role of the increased mitochondrial peroxide generation following denervation may be to stimulate axonal sprouting and regrowth [8] and the increase in Nox2 may also reflect a similar adaptive response. Nox2 has been shown to play a role in axonal growth cone dynamics and modulation of cytoskeletal proteins [39] and since this enzyme predominantly generates superoxide to the external face of the plasma or T-tubule membrane it is tempting to speculate that may play a role in muscle-nerve communication to modulate axonal re-growth or guidance [39].

To examine the effect of denervation on the antioxidant system response in skeletal muscle mitochondria, the contents of 3 major mitochondrial antioxidant enzymes, MnSOD, TRxR2 and GPx1 were analyzed. The MnSOD content in muscle did not change following denervation but our study focused on measuring protein content and not activity, therefore we cannot rule out altered activity. In contrast, both TrxR2 and GPx1 contents were significantly increased at 3 and 7 days post-denervation (Fig. 5A). The rapid increase in the content of these mitochondrial antioxidant enzymes may therefore also reflect an adaptive response following loss of innervation in an attempt to maintain redox homeostasis in the muscle.

We also hypothesized that the increased peroxide production following denervation would stimulate adaptive responses against oxidative damage and repair processes in an attempt to protect the muscle from degeneration. We observed that the Hsp70 content was increased following denervation and remained increased up to 21 days post-denervation (Supplementary data 2B) which is compatible with the possibility that the increased Hsp70 may play a role in protein homeostasis in the denervated muscle. Prolonged denervation of muscle also resulted in the increase content of Hsc70 (Fig. 5B).

Finally, we observed that the muscle Hsp25 content was also significantly increased from 3 days following denervation and remained increased until 21 days post-denervation (Fig. 5B). It has previously been shown that overexpression of Hsp25 in skeletal muscle cells produced dose-dependent protection against H_2_O_2_-induced damage that was associated with increased glutathione levels and GPx1 activity [40]. We did not observe any significant differences with time post-denervation in glutathione levels (Fig. 3B) but found a significant increase in the content of GPx1 which was maintained up to 21 days post denervation. Thus, the data are in accord with the possibility that the increased Hsp25 together with the changes in GPx1 are important in enhancing resistance to H_2_O_2_ damage in skeletal muscle fibers following denervation.

In summary, data indicate that following denervation, increased mitochondrial peroxide generation is evident at 7 days post-denervation and maintained up to 21 days post-denervation and that this increase is associated with a significant increase in the muscle content of various proteins involved in the potential generation of peroxides including Prx6 and cPLA2 which may be involved in the activation of NADPH oxidase. A significant increase was also seen in the content of several antioxidant enzymes and HSPs involved in the protection against oxidative damage, maintenance of redox homeostasis and proteostasis. These results support the possibility that, at least initially, the increase in peroxide production following denervation may stimulate adaptations to protect the muscle fibers; however sustained increased peroxide generation over the longer-term is likely to activate degenerative processes that lead to degeneration and muscle atrophy.

## Appendix A Supplementary data

Supplementary Data 1 (A) Representative western blots for p40^phox^ and (B) western blot analysis of p22^phox^, p47^phox^ and Rac-1 content in TA muscles from control mice (non-denervated) and mice at 1, 3, 7, 14 and 21 days post-denervation. Histograms represent the mean protein content and standard error of the mean for each experimental group. *p < 0.05 - **p < 0.01 - ***p < 0.001 compared with the control group. Supplementary Data 1

Supplementary Data 2 Representative western blots showing (A) Prx6 and (B) Hsp70 content in TA muscle from control mice (non-denervated) and mice at 1, 3, 7, 14 and 21 days post-denervation. Supplementary Data 2
