# Supplementary figures and images for: Redox responses in skeletal muscle following denervation
Source: Redox Biol. 2019 Aug 8;26:101294. doi: 10.1016/j.redox.2019.101294 (PMC6831873; doi:10.1016/j.redox.2019.101294)

## Slide 1
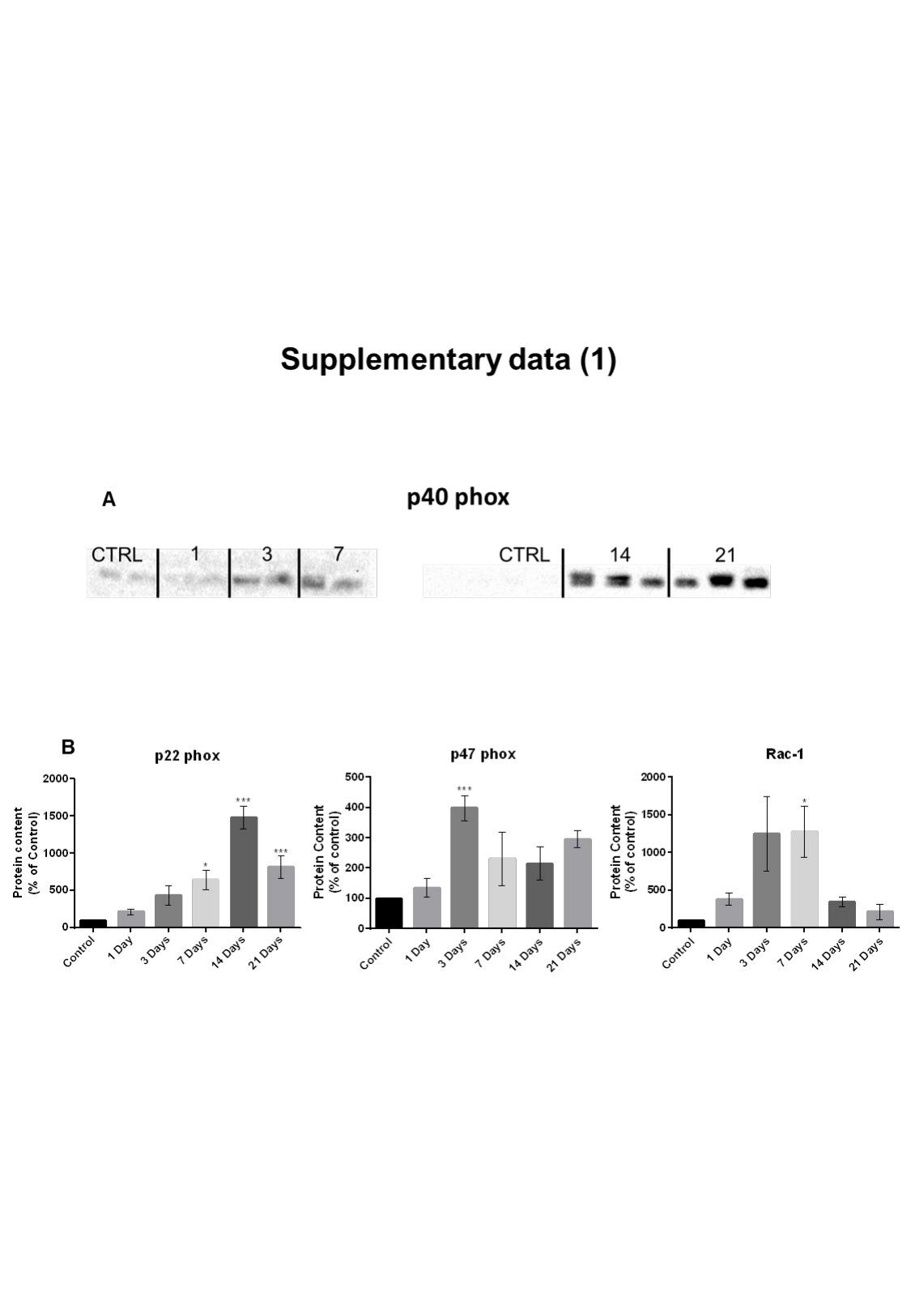

## Slide 2
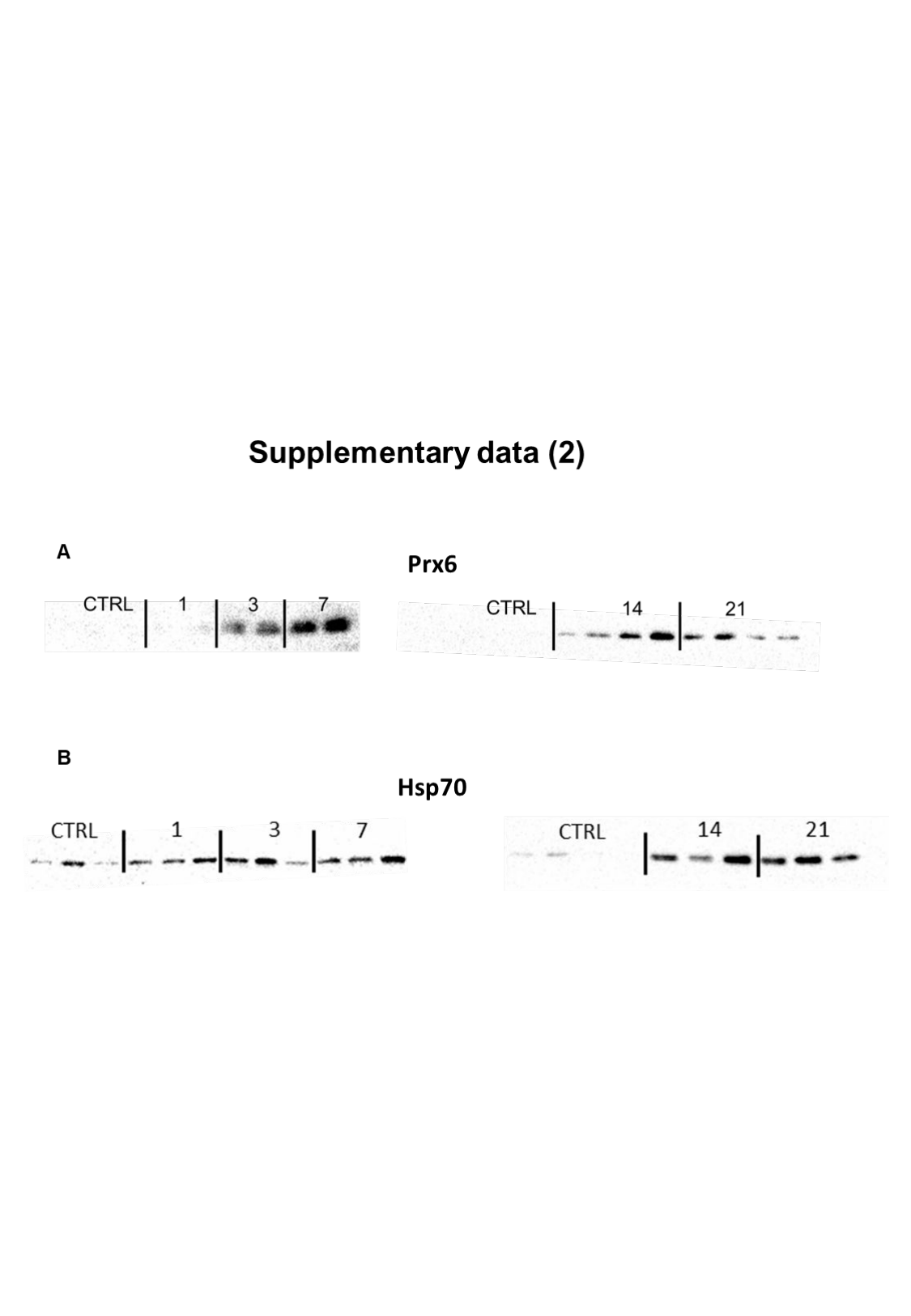

Supplement: Supplementary Data 1 — (A) Representative western blots for p40phox and (B) western blot analysis of p22phox, p47phox and Rac-1 content in TA muscles from control mice (non-denervated) and mice at 1, 3, 7, 14 and 21 days post-denervation. Histograms represent the mean protein content and standard error of the mean for each experimental group. *p < 0.05 - **p < 0.01 - ***p < 0.001 compared with the control group. [file mmc1.pptx]
